# Comparative Effects of Tadalafil Cream Versus Oral Tadalafil on Males with Erectile Disfunction Regarding Relationship Dynamics: A Secondary Analysis of Dyadic Adjustment Outcomes in a Randomized Crossover Trial

**DOI:** 10.3390/life15040668

**Published:** 2025-04-17

**Authors:** Dragoș-Mihail Trifu, Daniel-Corneliu Leucuța, Martina-Luciana Pintea-Trifu, Florin Elec, Nicolae Crișan, Dan Eniu, Ioan Coman

**Affiliations:** 1 Department of Urology, Iuliu Hațieganu University of Medicine and Pharmacy, 400012 Cluj-Napoca, Romania; trifu.dragos.mihail@elearn.umfcluj.ro (D.-M.T.); florinelec@elearn.umfcluj.ro (F.E.); drnicolaecrisan@elearn.umfcluj.ro (N.C.); coman_ioan55@yahoo.com (I.C.); 2 Department of Urology, Municipal Blaj Hospital, 515400 Blaj, Romania; 3 Department of Urology, Endoplus Clinic, 400165 Cluj-Napoca, Romania; 4 Department of Medical Informatics and Biostatistics, Iuliu Hațieganu University of Medicine and Pharmacy, 400349 Cluj-Napoca, Romania; 5 Department of Cellular and Molecular Biology, Iuliu Hațieganu University of Medicine and Pharmacy, 400349 Cluj-Napoca, Romania; pintea.trifu.martina@elearn.umfcluj.ro; 6 Clinical Institute of Urology and Renal Transplantation, Iuliu Hațieganu University of Medicine and Pharmacy, 400000 Cluj-Napoca, Romania; 7 Department of Urology, Municipal Cluj Hospital, Iuliu Hațieganu University of Medicine and Pharmacy, 400139 Cluj-Napoca, Romania; 8 Department of Surgical Oncology, Iuliu Hațieganu University of Medicine and Pharmacy, 400000 Cluj-Napoca, Romania; daneniu@elearn.umfcluj.ro

**Keywords:** erectile dysfunction, dyadic scale adjustment, relationship strength, tadalafil

## Abstract

Background/Objectives: Relationship quality is closely tied to sexual health. This study compared the effects of tadalafil cream and oral tadalafil on Dyadic Adjustment Scale (DAS) subscales and assessed the influence of age on treatment outcomes. Methods: This study includes a secondary analysis of data collected during a previously published randomized controlled crossover trial, but they were not published at that time. The participants (n = 35) completed both tadalafil cream and oral tadalafil interventions in a crossover design. Dyadic Adjustment, including DAS subscales, was assessed at baseline and after each intervention. Improvements across all DAS subscales were greater in the tadalafil cream group compared to the oral tadalafil group. Statistically significant differences were observed for Affective Expression (5.45, 95% CI: 0.22–10.67, *p* = 0.041) in the multivariate model. Notable gains were observed in Affective Expression and Dyadic Cohesion for the cream route. Within-group analysis showed statistically significant improvements in Affective Expression for both treatments and in Dyadic Cohesion for the cream route. The results show that younger participants benefited more from treatment, particularly in Affective Expression, Consensus subscales, and overall for Dyadic Adjustment. Conclusion: This study provides evidence that tadalafil intervention had a favorable impact on relationship dynamics, particularly in Affective Expression and Dyadic Cohesion.

## 1. Introduction

Erectile dysfunction is a public health issue with a significant impact on personal, couple, and social life, yet it is often insufficiently discussed or overlooked by men in the urologist’s office [1]. The psychological impact of such a diagnosis on the patient is frequently overlooked, particularly its harmful effect on intimate and couple relationships [2]. Erectile dysfunction is typically categorized into the following three etiological groups: organic, psychogenic, and mixed ED. This classification should be applied cautiously, as most cases are, in fact, of mixed etiology. Consequently, it has been recommended to use the terms “primary organic” or “primary psychogenic.” Anxiety and depression were also reported risk factors for erectile dysfunction [3]. Since erectile dysfunction is largely psychogenic, the urologist must consider its impact on the couple’s life during the medical interview [4,5].

As the first therapist the patient encounters, the urologist has a responsibility to guide the patient or couple toward psychotherapy, as sexual dysfunctions invariably affect couple dynamics [6].

For effective treatment, it is essential to establish a close collaboration between the urologist and the psychotherapist and to develop sexual health centers that address both the organic and psychological causes of sexual dysfunctions [7,8].

In evaluating erectile function, the IIEF-15 (International Index of Erectile Function) is a well-defined and validated tool used in numerous studies [9]. For assessing the quality of the couple’s relationship, the use of the Dyadic Adjustment Scale (DAS) is readily available to any clinician [10,11,12].

The Dyadic Adjustment Scale (DAS) is a widely recognized instrument for measuring the quality of adaptation between partners in marital or consensual dyadic relationships [13]. The DAS is extensively used in both clinical and research settings concerning couples [14]. With over 1000 scientific investigations employing the DAS, it has proven highly valuable in practice, and numerous studies have attested to its reliability and validity [15]. These studies demonstrate the utility of the DAS subscales in characterizing behavioral symptoms and issues within relationships caused by sexual dynamics disorders [16,17,18]. The diagnosis and treatment of these disorders (erectile dysfunction, premature ejaculation, delayed ejaculation, lack of sexual desire, or difficulties with orgasm) often require a multidisciplinary approach, involving urologists, psychotherapists, and sexual health specialists [4,14].

The DAS is recognized internationally, having been translated into languages such as French, German, Spanish, and Polish, among others. It is considered the most widely used tool for assessing marital quality globally [11].

In clinical settings, the DAS is primarily used to assess couples entering marital therapy or currently undergoing therapy [17,18,19,20]. Its multidimensional nature provides a comprehensive view of the relationship, aiding in treatment planning and identifying areas for discussion. Throughout therapy, the DAS serves as an objective measure for evaluating changes in partner interactions [19,21].

The DAS is suitable for both heterosexual and homosexual couples [22] without requiring any modifications for same-sex couples, although it should be noted that the instrument’s norms are based on heterosexual couples [17].

Previously, we undertook a randomized controlled crossover trial in which we compared oral treatment with topical administration of a phosphodiesterase type 5 inhibitor—tadalafil—demonstrating that the cream is non-inferior to oral administration in improving erectile function, as measured with IIEF-15 [23].

There are very few studies exploring the links between sexual function and marital satisfaction [24]. Furthermore, there are no studies, except our previously published study, comparing topical cream with oral phosphodiesterase type 5 inhibitors, and, implicitly, there are no studies assessing the DAS in these two groups.

Therefore, this study performed a secondary analysis aiming to evaluate the impact of tadalafil cream compared to oral tadalafil on relationship quality, as measured using the Dyadic Adjustment Scale (DAS) subscales, in participants from a previously conducted trial [23].

The objectives were as follows: to assess the difference in DAS subscales of different tadalafil administration routes; and to assess the difference between baseline and final values of DAS subscales.

## 2. Materials and Methods

### 2.1. Study Design and Setting

This study analyzed data collected during a previously published randomized controlled crossover trial (RCT), but they were not published at that time [23]. The previous published study aimed to evaluate the efficacy of tadalafil cream versus oral administration on erectile function domain score of IIEF-15 [23]. The original RCT, conducted at City Hospital “Prof. Dr. Ioan Puscas” and Regina Maria private practice was completed between 1 February 2022 and 1 September 2024 and involved 35 males meeting pre-specified inclusion criteria [23]. The current study utilizes data not published in the original trial to explore additional secondary endpoints, as variables of interest, expanding our understanding of couple dynamics provided by DAS subscales.

### 2.2. Participants

The participants in the original trial were recruited from a male population aged 18–75 years. The inclusion and exclusion criteria were detailed in the original RCT [23], and are briefly summarized here as follows:

Inclusion Criteria: Erectile dysfunction assessed by IEF-15-EF (erectile function) < 26, written informed consent and compliance with the study procedures, and agreement with the GDPR [25,26]

Exclusion Criteria: Severe cardiovascular, hepatic, renal, or hematological conditions; radiotherapy, pelvic surgery, and hormone therapy for prostate cancer; known hypersensitivity to any components of tadalafil (manufactered by Origin Pharma Distribution SRL, Voluntari City, Ilfov County, Romania) or Pentravan (simultaneous use of other medications for the management of erectile dysfunction, or drugs that could impact sexual function like beta-blockers or 5-α-reductase inhibitors; psychological or psychiatric disorders that could influence the study outcome; and PSA > 4 ng/mL [23].

All participants provided informed consent before participating in the original trial, and ethical approval was obtained from the Ethics Committee of Iuliu Hațieganu University of Medicine and Pharmacy, Cluj-Napoca (AVZ 14/3 February 2022). Each patient completed a questionnaire that included both the International Index of Erectile Function (IIEF-15) and the Dyadic Adjustment Scale (DAS).

### 2.3. Trial Interventions

The original RCT followed a crossover design in which each participant received both oral tadalafil and tadalafil cream in a randomized order, separated by a washout period of one week. This interval of time allows the drug to be eliminated, since the tadalafil half-life is 17.5 h, and the therapeutic effect lasts up to 36 h. Tadalafil cream, an investigational transdermal product, was applied locally to the penile meatus 10–15 min before intercourse, as per protocol. The cream was composed of tadalafil 0.5 g (manufactured by Fagron Hellas, 12 km N.R Trikala-Larissa, P.C.42100 Trikala, Greece), Ethoxydiglycol 0.3 g (manufactured by Fagron Hellas, 12 km N.R Trikala-Larissa, P.C.42100 Trikala, Greece), and liposomal Pentravan^®^ (manufactured by Fagron Hellas, 12 km N.R Trikala-Larissa, P.C.42100 Trikala, Greece). Each dose, delivered via an airless pen, is equivalent to a 20 mg tadalafil tablet. The cream features a user-friendly, non-greasy formulation optimized for comfort and safety, with a shelf life of 3 years. For comparison, oral tadalafil was administered at a standard 20 mg dose 30–60 min before intercourse. Each intervention period lasted two weeks, during which the participants adhered to the allocated tadalafil route administration.

### 2.4. Randomization and Blinding

Randomization was conducted using a computer-generated randomization schedule with a 1:1 allocation ratio, with blocks of 4 and 6 [27], with participants assigned to oral tadalafil or tadalafil cream as their starting condition. Blinding was not possible due to different modes of administration (oral vs. topical), with the participants being also the assessors by completing the questionnaires on their own. The procedures for randomization and blinding were described in detail in the primary publication [23]. Allocation concealment was ensured by having a separate individual, uninvolved in the study, handle randomization without accessing patient data.

### 2.5. Outcome Measures and Data Collection

The present secondary analysis investigates previously unreported outcome measures recorded during the original RCT. These include DAS subscales involving comparison between the tadalafil cream and oral tadalafil groups; and DAS subscales involving comparison before and after treatment for erectile dysfunction.

These data were collected at baseline, during each intervention period and post-intervention, as part of the original trial protocol. The collection methodologies were consistent with the procedures outlined in the original trial, ensuring uniformity and reliability across all outcome measures [23].

The DAS consists of 32 items and can be completed by one or both partners. Each item is scored based on a single response chosen from a list, with response anchors varying slightly depending on the question. The scale comprises the following four subscales: Dyadic Consensus (DC), Dyadic Satisfaction (DS), Dyadic Cohesion (DH), and Affective Expression (AE). A total adjustment score (DA) is calculated by summing the subscale scores. The scores from the profile forms are presented as T-scores, which are standardized, allowing each subscale to share the same mean and standard deviation. T-scores are categorized as follows: below 34 (moderately atypical, indicating a significant problem), 35–39 (mildly atypical, indicating a significant problem), 40–44 (slightly atypical, borderline, potential cause for concern), 45–55 (average, normal scores, no concerns), 56–60 (slightly atypical), 61–65 (mildly atypical), 66–70 (moderately atypical), and above 70 (markedly atypical) [15].

This scoring and classification system enables clinicians to identify areas of weakness as well as strengths in the relationship, guiding and recommending psychotherapy when appropriate. The DAS takes approximately 5–10 min to complete.

#### 2.5.1. Data Analysis

The data were analyzed using the R environment for statistical computing and graphics (R Foundation for Statistical Computing, Vienna, Austria), version 4.2.3 [28]. Continuous variables are presented as means with standard deviations. The reliability of the DAS was assessed with Cronbach’s alpha coefficient, the effect on the coefficient upon dropping each item, the correlation between each item, and the total score if items were dropped. Paired *t*-tests were used for within-subject comparisons due to the crossover design. Subgroup analyses were performed for those with ages above and below the median age value (51 years). For repeated measures across time points, a mixed-effects model with the subject as a random factor was applied to account for intra-individual variability, predicting the difference between final and baseline values of DAS subscales, with the treatment as the independent variable, adjusted for period, age ≥ 51 years, and with interaction between treatment and period. Model assumptions were checked, including the normality of the residuals (using quantile–quantile plots) and the presence of influential observations (using Cook’s distance). The model results were reported through effect sizes—coefficients, with 95% confidence intervals and *p*-values. Statistical significance was set at a *p*-value < 0.05.

#### 2.5.2. Handling Missing Data

All participants followed the protocol, and none were lost to follow-up, so there were no missing data. Therefore, no multiple imputations were needed to address this issue.

### 2.6. Ethical Considerations

All participants provided informed consent before participating in the original trial, and ethical approval was obtained from the Ethics Committee of Iuliu Hațieganu University of Medicine and Pharmacy, Cluj-Napoca (AVZ 14/3 February 2022) [23].

## 3. Results

The participant flow, randomization, and baseline characteristics for this secondary analysis are reported in the original trial article [23]. In brief, 35 participants were included and randomized, and they completed both intervention arms of the randomized crossover design (including a washout period of one week), offering complete observations for this secondary analysis (there were no participants lost to follow-up).

This secondary analysis focuses on additional outcomes measured but not reported in the primary publication, focusing on the DAS subscales.

### 3.1. DAS Reliability Assessment

The overall DAS Cronbach’s alpha was 0.91 (95% CI 0.86–0.95), indicating excellent reliability. The impact of each item removal on Cronbach’s alpha was assessed, and the impact was minimal, with the reliability of the scale maintaining a value between 0.90 and 0.91. Thus, no single item disproportionately affects the reliability of the scale. Most items have moderate to high correlations with the overall score, after the item was dropped, generally indicating good contribution to the overall construct.

### 3.2. DAS Subscale Comparison Between Tadalafil Cream and Oral Formulation

At baseline, the DAS subscale scores for participants in both the tadalafil cream and oral tadalafil groups were generally comparable (Table 1). However, slight differences were noted in some of the subscales. In the Dyadic Adjustment, Dyadic Cohesion, and Dyadic Satisfaction subscales, the oral tadalafil group showed slightly higher baseline scores in the first period and slightly lower baseline scores in the second period, compared to the tadalafil cream group. However, the differences are small, suggesting that the groups are sufficiently comparable.

The values observed for all DAS subscales improved more in the tadalafil-cream-receiving participants in the first period (Table 2). In the second period, the observed values of Affective Expression, Dyadic Adjustment, and Dyadic Satisfaction improved more in the oral-tadalafil-receiving participants, while Dyadic Cohesion improved more in the tadalafil-cream-receiving participants.

In the unadjusted analyses, on all participants, there were greater improvements in the tadalafil-cream-receiving participants for almost all DAS subscales, except for the Dyadic Satisfaction Scale, where the oral-tadalafil-receiving participants had greater improvements (Table 3).

In the subgroup analyses, in general, the participants aged below 51 years had improvements in all scores, and the improvements were greater compared to those observed in participants aged above 51 years. Those aged above 51 years had greater improvements when receiving tadalafil cream for almost all DAS subscales (the most pronounced being in the Affective Expression Scale), except for the Dyadic Cohesion Scale, where the oral-tadalafil-receiving participants had greater improvements. Those aged above 51 years had improvements in DAS subscales in about half of the subscales and declines in DAS subscales in about half of the subscales. In this subgroup, the most important improvement for the tadalafil-cream-receiving participants was for Dyadic Cohesion, compared to the oral-tadalafil-receiving participants. Also, Dyadic Satisfaction improved more in the oral-tadalafil-receiving participants compared to tadalafil-cream-receiving participants.

**Figure 1:**
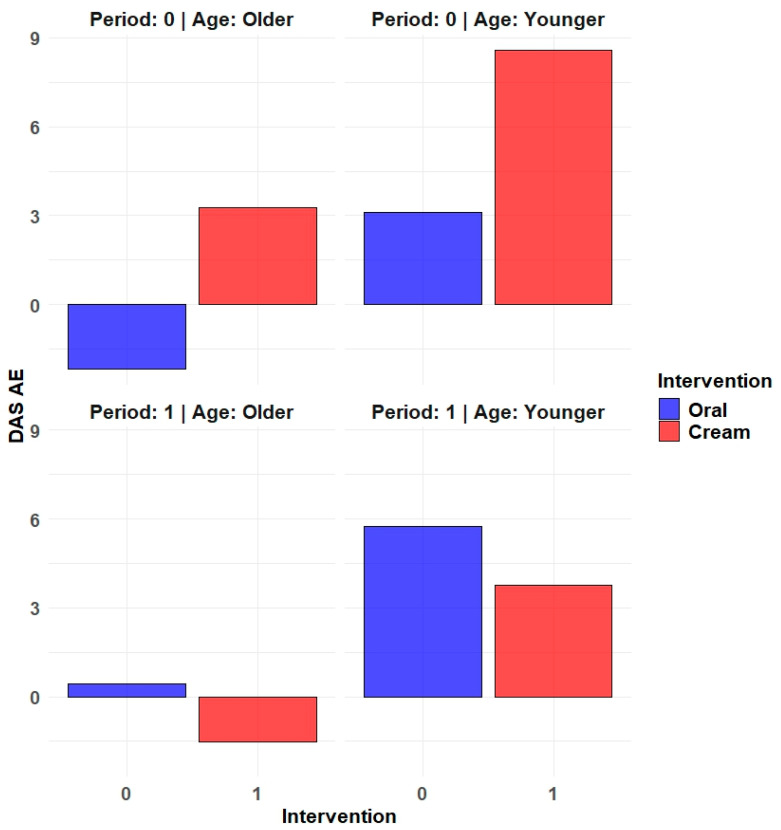
DAS AE subscale predicted mean change (final minus baseline values) with linear mixed models, using treatment (tadalafil cream vs. oral tadalafil), and adjusted for period and age ≥ 51 years.

However, in the multivariate analysis, using linear mixed models, after adjustment for the study period, age ≥ 51 years, and with an interaction term between treatment and period, for all the DAS subscales, there were greater improvements in the tadalafil cream group compared to oral tadalafil group (Table 4). For the Affective Expression scale, the result was statistically significant; while, for Dyadic Adjustment, Consensus, and Cohesion, the *p*-values were around 0.10; and, for Dyadic Satisfaction, the *p*-value was 0.459. The most important improvements were observed for Affective Expression (Figure 1), followed by Dyadic Cohesion. Those aged above 51 years had decreases in all DAS subscales, with the decrease being statistically significant for Affective Expression, Consensus subscales, and for the overall Dyadic Adjustment. No significant period effects were observed, and no significant interactions between treatment and period were observed.

The Cohen d effect sizes for the linear mixed models are presented in Table 5. Moderate effect sizes were observed concerning intervention for Affective Expression, Dyadic Consensus, and Dyadic Adjustment, and low effect sizes for the other scales. Concerning age ≥ 51 years, a large effect size was observed for Affective Expression; moderate effect sizes were observed for Dyadic Consensus, Dyadic Satisfaction, and Dyadic Adjustment; and a low effect size was observed for Dyadic Cohesion.

### 3.3. Evolution Between Baseline and Final Values

Finally, we delved into the effects of the interventions between the baseline and final values of each DAS subscale.

Regarding all participants, for oral and cream administration on all DAS subscales (except for Dyadic Consensus within the oral administration), the observed final values had higher means compared to the baseline values (Table 6). The differences were statistically significant for Affective Expression for both administration routes. As for Dyadic Cohesion, there was a significant difference for the cream administration route only.

In the subgroup analyses, the participants aged below 51 years in the tadalafil-cream-receiving group had significant improvements in scores, concerning Affective Expression, while, for those aged above 51 years, there were no significant differences. Nevertheless, there were several instances where the results were close to the significance threshold.

## 4. Discussion

The findings of this cross-over randomized controlled trial secondary analysis suggest that, although improvements across all Dyadic Adjustment Scale (DAS) subscales were generally greater in the tadalafil cream group compared to the oral tadalafil group, and these differences reached statistical significance only for Affective Expression. The Affective Expression Dyadic Cohesion subscales showed the most gains, suggesting that tadalafil cream may have a greater influence on relationship cohesiveness and emotional expression. Tadalafil cream generally showed greater improvements in DAS subscales compared to oral tadalafil, particularly in younger participants (<51 years). In older participants (≥51 years), the changes were smaller and showed no consistent pattern favoring one formulation. For all DAS subscales, the improvements were diminished by older age, with the effect being statistically significant for Affective Expression, Consensus subscales, and for the overall Dyadic Adjustment. There were improvements in DAS subscales between the baseline and final values, reaching statistical significance for Affective Expression (for both routes) and for Dyadic Cohesion (within the cream route).

Affective Expression and Dyadic Cohesion showed the most gains among the DAS subscales, especially in the tadalafil cream group. This implies that relationship quality factors, including emotional connection and cooperation, may be more strongly impacted by tadalafil cream. Greater marital cohesiveness and expressiveness may be fostered by the cream’s localized administration and less systemic side effects, which may boost comfort and emotional connection during intimate encounters.

For both therapy groups, improvements in the Affective Expression and Dyadic Cohesion subscales were statistically significant when comparing baseline and end values. Interestingly, similar improvements were seen in both delivery methods, highlighting the possible advantages of tadalafil, whether taken orally or as a cream, for relationship cohesion and emotional expressiveness.

Beyond its primarily physical effects, this research demonstrates the therapeutic influence of tadalafil on marital dynamics, indicating its potential to improve emotional and relational well-being.

Tadalafil cream showed larger improvements in DAS subscales compared to Oral Tadalafil, with the results being especially noticeable in younger participants (<51 years), where improvements in Affective Expression, Consensus, and overall Dyadic Adjustment were more substantial. On the other hand, older individuals (≥51 years) showed no discernible trend favoring either formulation and smaller variations across subscales. Significant effects of age were also seen, with older participants showing statistically significant declines in Affective Expression, Consensus, and total Dyadic Adjustment. These results imply that tadalafil cream may have a stronger impact on younger participants, whilst its benefits on older participants may be lessened due to physiological or relational aspects associated with aging and relationship duration.

Our rationale for examining DAS in this investigation lies in the growing recognition that the dynamics of the relationship are associated with the experience of ED. ED is not simply a physiological disorder of a separated individual, but also contains significant interpersonal elements. While tools such as the Female Sexual Function Index and Hospital Anxiety and Depression Scale can capture some aspects of sexual function and mental state, for the partner or for the patient, they do not directly assess the quality of the relationship (which is the core of DAS).

The literature is extremely limited on this topic, with only a couple of studies related to DAS and PDE5i. One study showed no significant improvement over DAS subscales on men presented with ED and treated with PDE5i; however, for women, it indicates that a fulfilling and positive overall relationship serves as a strong foundation for sexual enhancement, whereas, for men, sexual difficulties and relationship issues seem to be more intricately interconnected [29]. One trial (The South Australian Couples Sildenafil Study = SACS) found that, overall, Dyadic Satisfaction levels were maintained in the active treatment with sildenafil group but declined in the placebo group. Also, it demonstrated that ED had no effect on relationship status measured using the DAS, although there might have been a ceiling effect [30]. There were no studies found comparing PDE5i oral and topical formulations effects on DAS.

Protocol adherence is an important part of trials. Due to the sensitive topic—the intrameatal administration of the drug—of the study, the adherence could not be directly measured. From a methodological point of view, a direct measurement would interfere with the intervention. Nevertheless, since the participants were in real need of help for their erectile disfunction, it is likely that they followed the protocol to improve their sex lives. The participants did not have any financial incentive for the study. The only incentive was their sex life problems, which may be particularly relevant for younger individuals due to their more active sexual life and emotionally intense relationships. Additionally, the absence of comorbidities and optimal biological status in younger subjects may contribute to a greater likelihood of positive treatment response [31].

The literature addresses several concerns about males with ED. One of them is the discrepancy between patients’ own assessment of erection rigidity, intercourse duration, and professional findings that show that the male population has a lower perception of virility and sexual function; therefore, thoughtful counseling about treatment goals and expectations becomes mandatory [32,33]. In this population, psychotherapy or sex therapy are critical to ensure success [34]. Some studies revealed that the combination of PDE-5 inhibitors with psychological interventions demonstrates significant potential in the management of psychogenic erectile dysfunction [35,36]. However, no definitive conclusions can be drawn regarding the superiority of one psychological intervention over another, and larger-scale studies are required to validate these preliminary findings [37,38]. Moreover, the combination of tadalafil with behavioral therapy and psychological counseling has been shown to be more effective than tadalafil monotherapy, leading to greater therapeutic satisfaction, improved sexual quality of life, and enhanced partner relationships [36,39].

Some authors have pointed out that there is a new concept emerging in the literature, as follows: precarious manhood beliefs (PMB) and their relationship with erectile dysfunction (ED) in cisgender men remains unclear. Since erectile function is often perceived as a core aspect of masculinity, ED may be interpreted as a failure, threatening a man’s self-image and psychological well-being. While causality requires further investigation, these findings suggest that higher PMB endorsement may contribute to increased performance-related anxiety, elevating psychological distress and the risk of developing ED [40,41].

It seems like mindfulness has a beneficial impact on ED, however, randomized studies with active control groups are required in order to establish the benefits of this kind of sexual-therapy-based intervention [42].

A summarization of previous studies of comparisons of tadalafil with sildenafil and Vardenafil or combination therapy (PDE5i + cognitive behavioral therapy) is presented in Table 7. The current literature indicates significant patient and partner preferences for PDE5 inhibitors for erectile dysfunction (ED), with tadalafil as the agent of choice on account of the long duration of action, flexible dosing interval, and simplicity of use—most significantly among younger men and female partners. While all agents are effective for improving erectile function, convenience and lifestyle compatibility are key to compliance and satisfaction to such an extent that many patients switch to tadalafil with time. Compliance for long-term periods is associated with greater quality of life and indirectly with improved relational dynamics, though direct relational measures were not typically of primary interest. Significantly, adjunct treatment with psychotherapy in the form of cognitive behavioral therapy was found to offer greater improvement in both erectile function and relational satisfaction compared with pharmacologic treatment alone.

### 4.1. Study Limitations

This study’s limitations include its secondary analysis design, which may lack the power to detect small but meaningful effects, as the original trial was not specifically focused on relationship outcomes. Additionally, self-reported DAS scores may be subject to bias, and the modest sample size limits generalizability [18,19,20]. The small sample size is another limitation. Nevertheless, the cross-over design is particularly advantageous, since the comparisons are intrasubject, and the power to detect changes is high even for small sample sizes, compared to parallel designs. The large age range (18–75 years), although important for generalizability, poses another limitation of this study, superposed on the sample size. A wide age range introduces heterogeneity in physiological, psychological, and relational factors that may influence the outcomes and treatment response through increased variability. To address this limitation, we performed stratified analyses by age, and the multivariate model included age as a confounding variable. The lack of blinding increases the risk of performance and detection bias. Unfortunately, the use of two different administration routes precludes masking in this case. Residual confounding cannot be ruled out, and variables like relationship duration and quality might have influenced the observed results. Participant adherence is difficult to verify in this context. Nevertheless, the motivation to solve their problem of erectile disfunction makes nonadherence improbable. We acknowledge that the absence of biological (hormonal evaluations like testosterone, cortisol, prolactin, and penile Doppler) and physiological correlations (heart rate variability, as a marker of autonomous nervous system) limits our ability to fully elucidate the mechanisms underlying the treatment response. The absence of partner-reported feedback, in the form of Likert-scales like DAS outcomes, as well as of qualitative testimonials to provide a more complete view of relationship dynamics, is another limitation of this study. Also, other psychological factors can provide a more thorough view on the topic. Future research with larger samples, specifically aimed at relational outcomes of tadalafil formulations, is needed to explore this topic more in depth.

### 4.2. Study Strengths

This study is the first to assess DAS subscales regarding tadalafil administration, offering unique insights into how different formulations may impact relationship dynamics. Moreover, it is one of the few trials on PDE5i assessing their effect on DAS. The crossover design, where each participant serves as their own control, reduces variability and enhances this study’s power. The use of a validated instrument provides a comprehensive and reliable assessment. Adjustments for the study period and interaction terms further strengthen the analysis.

### 4.3. Future Directions

Future research should focus on larger, dedicated trials to more robustly assess the effects of tadalafil formulations on relationship quality, with a particular emphasis on Dyadic Adjustment Scale (DAS) subscales like Affective Expression and Dyadic Cohesion, which showed the most promise in this study. Expanding the assessment to include additional measures of relational well-being and conducting long-term follow-ups could provide deeper insights into the sustained impact of tadalafil on relationship dynamics. A mediation model testing whether IIEF-15 improvements predict DAS improvements could strengthen causal claim.

In future, larger-scale investigations, classifying “responders” based on both treatment satisfaction and ED improvement, could help clarify whether relationship gains stem from symptom relief, while examining baseline factors (e.g., age, ED severity, and relationship quality) may further identify which patients stand to benefit most from ED interventions.

Addressing the psychological distress, performance anxiety, and the impact of ED on couple’s dynamics, as well as cognitive behavioral therapy (CBT), mindfulness-based interventions, and psychosexual counseling, may enhance treatment efficacy and long-term adherence. Furthermore, the combination of PDE-5 inhibitors with psychotherapy has shown superior therapeutic outcomes, resulting in greater patient satisfaction and improved couple relationships.

Future research should aim to compare different psychotherapeutic interventions, investigate ED treatment in a broader and more stratified population (including men with varying comorbidities and psychological profiles), assess the durability of treatment outcomes over extended periods to determine the long-term efficacy of combined therapy, and explore the role of precarious manhood beliefs (PMB) and their impact on treatment success, as well as how interventions targeting masculinity-related stress may improve sexual function and emotional well-being. In order to achieve this, large-scale randomized controlled trials are warranted in order to evaluate the long-term effectiveness of psychotherapy in ED treatment, particularly in combination with pharmacological approaches.

#### Clinical Implications

The need for a multidisciplinary team approach that involves both partners in the treatment of sexual health issues is evident, given the significant impact of these issues on relationships. The increasing prevalence of this disorder among younger males further highlights the necessity for specialized medical centers where urologists, psychotherapists, and sexual health specialists collaborate to provide holistic care.

## 5. Conclusions

This study provides evidence that tadalafil intervention had a favorable impact on relationship dynamics, particularly in Affective Expression and Dyadic Cohesion. In the multivariate analysis, tadalafil cream, compared to the oral formulation, had greater improvements overall; moreover, on all subscales, the results were significant for the Affective Expression subscale. For most DAS subscales, both oral and cream administration resulted in significantly higher final mean values compared to the baseline. The results show that younger participants benefited more from treatment, particularly in Affective Expression, Consensus subscales, and, overall, for Dyadic Adjustment, while older participants experienced diminished and inconsistent improvements, highlighting age as a key factor in treatment effectiveness.

## Figures and Tables

**Table 1:** DAS subscale comparison between tadalafil cream and oral formulation at the beginning and the end of each study period.

	Period 1				Period 2			
DAS, Mean (SD)	Baseline		Final		Baseline		Final	
	Tadalafil Cream	Oral Tadalafil	Tadalafil Cream	Oral Tadalafil	Tadalafil Cream	Oral Tadalafil	Tadalafil Cream	Oral Tadalafil
	(n = 18)	(n = 17)	(n = 18)	(n = 17)	(n = 17)	(n = 18)	(n = 17)	(n = 18)
Affective Expression	47.56 (10.79)	47.29 (6.75)	52.89 (9.02)	48.24 (7.49)	49.12 (7.59)	48.83 (10.72)	50.71 (6.68)	51.33 (8.48)
Dyadic Consensus	55.22 (15.1)	56 (7.38)	56.78 (12.11)	56.06 (7.55)	57.29 (7.1)	55.28 (14.81)	57.24 (6.97)	55.06 (13.88)
Dyadic Cohesion	49.67 (12.62)	53.82 (11.63)	52.89 (8.98)	54.29 (9.95)	52.24 (11.05)	50.33 (13.43)	53.12 (10.9)	50.28 (13.46)
Dyadic Satisfaction	50.83 (10.04)	54.88 (8.08)	52.61 (5.62)	55.88 (7.87)	56.53 (7.33)	51.44 (10.13)	56.29 (8.1)	52.89 (6.99)
Dyadic Adjustment	52.78 (12.98)	55.47 (5.49)	55.28 (9.06)	56.06 (6.21)	56.53 (5.77)	52.83 (13.39)	56.88 (5.78)	53.67 (11.14)

DAS, Dyadic Adjustment Scale; SD, standard deviation.

**Table 2:** DAS subscale change (final minus baseline values) by study period for tadalafil cream and oral formulations.

	First Period		Second Period	
DAS, Mean (SD)	Tadalafil Cream	Oral Tadalafil	Tadalafil Cream	Oral Tadalafil
	(n = 18)	(n = 17)	(n = 17)	(n = 18)
Affective Expression	5.33 (12.78)	0.94 (3.27)	1.59 (6.15)	2.5 (6.26)
Dyadic Consensus	1.56 (5.76)	0.06 (1.03)	−0.06 (0.97)	−0.22 (3.72)
Dyadic Cohesion	3.22 (6.58)	0.47 (4.57)	0.88 (2.5)	−0.06 (6.55)
Dyadic Satisfaction	1.78 (6.59)	1 (4.26)	−0.24 (2.66)	1.44 (4.76)
Dyadic Adjustment	2.5 (6.44)	0.59 (2.45)	0.35 (1.66)	0.83 (5.06)

DAS, Dyadic Adjustment Scale; SD, standard deviation.

**Table 3:** DAS subscale change (final minus baseline values) observed in all subjects for tadalafil cream and oral formulations, and separately for participants aged above and below the median age.

DAS Change, Mean (SD)	Tadalafil Cream (n = 35)	Oral Tadalafil (n = 35)	Difference (95% CI)
All			
Affective Expression	3.51 (10.16)	1.74 (5.03)	1.77 (−1.1–4.64)
Dyadic Consensus	0.77 (4.21)	−0.09 (2.73)	0.86 (−0.02–1.74)
Dyadic Cohesion	2.09 (5.1)	0.2 (5.6)	1.89 (−0.53–4.3)
Dyadic Satisfaction	0.8 (5.11)	1.23 (4.46)	−0.43 (−1.8–0.94)
Dyadic Adjustment	1.46 (4.82)	0.71 (3.95)	0.74 (−0.32–1.8)
Age ≥ 51 years	(n = 18)	(n = 18)	
Affective Expression	0.06 (7.91)	0.78 (3.81)	−0.72 (−4.45–3.01)
Dyadic Consensus	−0.44 (3.43)	−0.94 (3.24)	0.5 (−0.34–1.34)
Dyadic Cohesion	2.44 (5.03)	−1.28 (5.78)	3.72 (−0.42–7.86)
Dyadic Satisfaction	−0.89 (2.72)	1.11 (4.19)	−2 (−4.29–0.29)
Dyadic Adjustment	0.06 (3.33)	−0.22 (3.69)	0.28 (−1.11–1.66)
Age < 51 years	(n = 17)	(n = 17)	
Affective Expression	7.18 (11.18)	2.76 (6.01)	4.41 (−0.02–8.85)
Dyadic Consensus	2.06 (4.66)	0.82 (1.7)	1.24 (−0.44–2.91)
Dyadic Cohesion	1.71 (5.3)	1.76 (5.11)	−0.06 (−2.57–2.45)
Dyadic Satisfaction	2.59 (6.4)	1.35 (4.86)	1.24 (0.04–2.43)
Dyadic Adjustment	2.94 (5.74)	1.71 (4.09)	1.24 (−0.51–2.98)

DAS, Dyadic Adjustment Scale; SD, standard deviation; CI, confidence interval.

**Table 4:** DAS subscale change (final minus baseline values) predicted with linear mixed models, using treatment (tadalafil cream vs. oral tadalafil), and adjusted for period, age ≥ 51 years, and with an interaction term between treatment and period, and using random effects for participants.

DAS Predicted Subscale	Treatment (95% CI)	*p*	Period (95% CI)	*p*	Age ≥ 51 Years (95% CI)	*p*	Treatment:Period (95% CI)	*p*
Affective Expression	5.45 (0.22–10.67)	0.041	2.61 (−2.61–7.84)	0.321	−5.29 (−9.67–−0.91)	0.019	−7.41 (−16.18–1.35)	0.096
Dyadic Consensus	1.97 (−0.36–4.3)	0.097	0.19 (−2.14–2.52)	0.871	−2.36 (−4.55–−0.18)	0.035	−2.28 (−6.64–2.09)	0.302
Dyadic Cohesion	3.03 (−0.64–6.7)	0.104	−0.25 (−3.92–3.42)	0.892	−1.39 (−4.2–1.42)	0.327	−2.37 (−7.99–3.25)	0.403
Dyadic Satisfaction	1.21 (−2.04–4.47)	0.459	0.88 (−2.38–4.14)	0.591	−2.19 (−5.16–0.78)	0.145	−3.33 (−9.27–2.61)	0.267
Dyadic Adjustment	2.46 (−0.45–5.37)	0.096	0.79 (−2.12–3.71)	0.588	−2.75 (−5.49–−0.02)	0.048	−3.49 (−8.96–1.98)	0.207

DAS, Dyadic Adjustment Scale; CI, confidence interval.

**Table 5:** The Cohen D effect sizes for DAS subscale change (final minus baseline values) predicted with linear mixed models, using treatment (tadalafil cream vs. oral tadalafil), and adjusted for period, age ≥ 51 years, and with an interaction term between treatment and period, using random effects for participants.

	Intervention	Period	Age ≥ 51 Years	Treatment: Period
Affective Expression	0.56	0.27	−0.85	−0.6
Dyadic Consensus	0.53	0.05	−0.76	−0.37
Dyadic Cohesion	0.42	−0.03	−0.35	−0.3
Dyadic Satisfaction	0.22	0.16	−0.52	−0.4
Dyadic Adjustment	0.53	0.17	−0.71	−0.45

**Table 6:** DAS evolution between baseline and final values, for each treatment, overall, and separately, for participants aged above and below the median age.

DAS, Mean (SD)	Baseline	Final	Difference (95% CI)	*p*-Value
Oral (n = 35)				
Affective Expression	48.09 (8.92)	49.83 (8.05)	1.74 (0.02; 3.47)	0.047
Dyadic Consensus	55.63 (11.64)	55.54 (11.11)	−0.09 (−1.02; 0.85)	0.854
Dyadic Cohesion	52.03 (12.53)	52.23 (11.89)	0.2 (−1.72; 2.12)	0.834
Dyadic Satisfaction	53.11 (9.22)	54.34 (7.48)	1.23 (−0.3; 2.76)	0.112
Dyadic Adjustment	54.11 (10.27)	54.83 (9.03)	0.71 (−0.64; 2.07)	0.293
Oral, age ≥ 51 years (n = 18)				
Affective Expression	51.11 (9.16)	51.89 (8.78)	0.78 (−1.12; 2.67)	0.399
Dyadic Consensus	58 (8.32)	57.06 (8.97)	−0.94 (−2.56; 0.67)	0.234
Dyadic Cohesion	52.11 (12.9)	50.83 (13.4)	−1.28 (−4.15; 1.6)	0.361
Dyadic Satisfaction	54 (6.1)	55.11 (6.04)	1.11 (−0.97; 3.19)	0.276
Dyadic Adjustment	55.89 (6.84)	55.67 (7.78)	−0.22 (−2.06; 1.61)	0.801
Oral, age < 51 years (n = 17)				
Affective Expression	44.88 (7.66)	47.65 (6.78)	2.76 (−0.32; 5.85)	0.076
Dyadic Adjustment	52.24 (12.94)	53.94 (10.37)	1.71 (−0.4; 3.81)	0.105
Dyadic Consensus	53.12 (14.19)	53.94 (13.09)	0.82 (−0.05; 1.7)	0.064
Dyadic Cohesion	51.94 (12.52)	53.71 (10.26)	1.76 (−0.86; 4.39)	0.173
Dyadic Satisfaction	52.18 (11.81)	53.53 (8.87)	1.35 (−1.15; 3.85)	0.268
Cream (n = 35)				
Affective Expression	48.31 (9.27)	51.83 (7.93)	3.51 (0.03; 7)	0.048
Dyadic Consensus	56.23 (11.79)	57 (9.81)	0.77 (−0.67; 2.22)	0.286
Dyadic Cohesion	50.91 (11.78)	53 (9.81)	2.09 (0.33; 3.84)	0.021
Dyadic Satisfaction	53.6 (9.17)	54.4 (7.08)	0.8 (−0.96; 2.56)	0.361
Dyadic Adjustment	54.6 (10.18)	56.06 (7.58)	1.46 (−0.2; 3.11)	0.082
Cream, age ≥ 51 years (n = 18)				
Affective Expression	51.89 (8.22)	51.94 (9.63)	0.06 (−3.88; 3.99)	0.977
Dyadic Consensus	57.39 (8.51)	56.94 (9.07)	−0.44 (−2.15; 1.26)	0.590
Dyadic Cohesion	50.22 (9.94)	52.67 (9.39)	2.44 (−0.06; 4.95)	0.055
Dyadic Satisfaction	54.83 (5.7)	53.94 (6.27)	−0.89 (−2.24; 0.46)	0.184
Dyadic Adjustment	55.67 (6.94)	55.72 (7.47)	0.06 (−1.6; 1.71)	0.944
Cream, age < 51 years (n = 17)				
Affective Expression	44.53 (9.01)	51.71 (5.91)	7.18 (1.43; 12.92)	0.018
Dyadic Consensus	55 (14.67)	57.06 (10.83)	2.06 (−0.34; 4.45)	0.087
Dyadic Cohesion	51.65 (13.74)	53.35 (10.52)	1.71 (−1.02; 4.43)	0.203
Dyadic Satisfaction	52.29 (11.85)	54.88 (8.01)	2.59 (−0.7; 5.88)	0.115
Dyadic Adjustment	53.47 (12.89)	56.41 (7.9)	2.94 (−0.01; 5.89)	0.051

DAS, Dyadic Adjustment Scale; SD, standard deviation; CI, confidence interval.

**Table 7:** Comparisons of phosphodiesterase 5 inhibitors.

Study/Reference	Therapy/PDE5i	Sample/Design	Relationship or ED Measures	Key Findings
Raheem & Kell et al., 2009 [43]	Sildenafil, Vardenafil, and tadalafil	n = 60 men with non-organic ED; randomized controlled trial	IIEF; patient and partner satisfaction (custom questionnaires)	Patients who tried all three drugs generally reported better satisfaction when given the choice. The study highlights differences in preference among PDE5 inhibitors.
Gong et al., 2011 [44]	Tadalafil	Survey-based evaluation in men with ED (and partner feedback)	IIEF; partner-reported satisfaction	Both patients and their female partners significantly preferred tadalafil due to its flexible dosing window and perceived ease of use.
Rubio-Aurioles et al., 2013 [45]	PDE5 inhibitors (various)	Prospective observational study in Latin American men with ED	IIEF; treatment persistence/adherence data (indirectly linked to relationship outcomes)	Reported adherence patterns over 6 months. The findings underscore the importance of sustained treatment for long-term satisfaction, though direct relationship measures were not the focus.
Ströberg P et al., 2003 [46]	Transition from sildenafil to tadalafil	European multicenter, open-label, crossover study in men with ED	IIEF; patient-reported preference	The majority of patients switched from sildenafil to tadalafil, indicating a clear preference for the latter, based on improved convenience and satisfaction.
Mirone V et al., 2009 [47]	Comparison among tadalafil, Vardenafil, and sildenafil	Narrative review of multiple patient preference studies	IIEF; satisfaction and preference metrics	Review indicates that, while all agents improve ED, differences in onset, duration, and side effects may drive preference—young men often favor tadalafil.
Pyrgidis N et al., 2021 [48]	Tadalafil	Meta-analysis of randomized controlled trials in men with ED	IIEF; safety and efficacy outcomes	Confirmed that tadalafil is effective and safe for treating ED, with benefits reflected in both erectile function and patient-reported satisfaction.
Rubio-Aurioles et al., 2006 [49]	Sildenafil vs. Vardenafil	Double-blind, randomized, crossover study in men with ED	IIEF	Vardenafil demonstrated nominal statistical superiority over sildenafil across several commonly used efficacy measures, while both medications were generally well tolerated.
Zhang et al., 2020 [35]	Sildenafil + brief psychodynamic psychotherapy	n = 63 men with non-organic ED; Randomized controlled trial	IIEF; marital/relationship satisfaction (custom questionnaires)	Combination therapy produced significantly better improvements in erectile function and relationship satisfaction compared to PDE5i alone.
Atallah et al., 2021 [38]	PDE5i (various) + psychological interventions (systematic review)	Systematic review of 8 studies (mixed designs)	IIEF; partner satisfaction; other psychometric scales	Studies indicated that adding psychotherapy (e.g., CBT) to PDE5i therapy enhanced improvements in ED severity and relationship functioning compared to PDE5i monotherapy.
Elterman et al., 2021 [7]	PDE5i (sildenafil/Vardenafil/tadalafil)	Narrative review of clinical and economic burden	ED questionnaires (IIEF-5/15); QoL measures (SF-36)	This review highlights the significant quality of life and economic burden of ED, underscoring the need for greater awareness and improved access to effective treatments.
Althof et al., 2023 [37]	PDE5i + psychosexual counseling	Commentary/expert opinion	Focus on integrated ED management	Emphasizes that combining PDE5i with counseling may lead to superior couple outcomes, although data comparing specific PDE5i on relationship metrics are limited.

## Data Availability

The dataset is available upon request from the authors.
